# Effect of neutron beam properties on dose distributions in a water phantom for boron neutron capture therapy

**DOI:** 10.1093/jrr/rrae076

**Published:** 2024-10-04

**Authors:** Akihisa Ishikawa, Hiroki Tanaka, Satoshi Nakamura, Hiroaki Kumada, Yoshinori Sakurai, Kenichi Watanabe, Sachiko Yoshihashi, Yuki Tanagami, Akira Uritani, Yoshiaki Kiyanagi

**Affiliations:** Research Group for Nuclear Sensing, Nuclear Science and Engineering Center, Japan Atomic Energy Agency, 2-4 Shirakata, Tokai-mura, Ibaraki 319-1195, Japan; Department of Applied Energy, Graduate School of Engineering, Nagoya University, Furo-cho, Chikusa-ku, Nagoya, Aichi 464-8603, Japan; Integrated Radiation and Nuclear Science, Kyoto University, 2, Asashiro-Nishi, Kumatori-cho, Sennan-gun, Osaka 590-0494, Japan; Division of Radiation Safety and Quality Assurance, National Cancer Center Hospital, 5-1-1, Tsukiji, Chuo-ku, Tokyo 104-0045, Japan; Proton Medical Research Center, Institute of Medicine, University of Tsukuba, 1-1-1, Tennodai, Tsukuba, Ibaraki 305-8575, Japan; Integrated Radiation and Nuclear Science, Kyoto University, 2, Asashiro-Nishi, Kumatori-cho, Sennan-gun, Osaka 590-0494, Japan; Department of Applied Quantum Physics and Nuclear Engineering, Kyushu University, 744, Motooka, Nishi-ku, Fukuoka 819-0395, Japan; Department of Applied Energy, Graduate School of Engineering, Nagoya University, Furo-cho, Chikusa-ku, Nagoya, Aichi 464-8603, Japan; Department of Applied Energy, Graduate School of Engineering, Nagoya University, Furo-cho, Chikusa-ku, Nagoya, Aichi 464-8603, Japan; Department of Applied Energy, Graduate School of Engineering, Nagoya University, Furo-cho, Chikusa-ku, Nagoya, Aichi 464-8603, Japan; Hokkaido University, Kita-13 Nishi-8, Kita-ku, Sapporo, Hokkaido 060-8628, Japan

**Keywords:** boron neutron capture therapy, beam properties, thermal/epithermal ratio, fast neutron component, γ-ray component, skin dose

## Abstract

From the viewpoints of the advantage depths (ADs), peak tumor dose and skin dose, we evaluated the effect on the dose distribution of neutron beam properties, namely the ratio between thermal and epithermal neutron fluxes (thermal/epithermal ratio), fast neutron component and *γ*-ray component. Several parameter surveys were conducted with respect to the beam properties of neutron sources for boron neutron capture therapy assuming boronophenylalanine as the boron agent using our dose calculation tool, called SiDE. The ADs decreased by 3% at a thermal/epithermal ratio of 20–30% compared with the current recommendation of 5%. The skin dose increased with the increasing thermal/epithermal ratio, reaching a restricted value of 14 Gy_eq_ at a thermal/epithermal ratio of 48%. The fast neutron component was modified using two different models, namely the ‘linear model’, in which the fast neutron intensity decreases log-linearly with the increasing neutron energy, and the ‘moderator thickness (MT) model’, in which the fast neutron component is varied by adjusting the MT in a virtual beam shaping assembly. Although a higher fast neutron component indicated a higher skin dose, the increment was <10% at a fast neutron component of <1 × 10^−12^ Gy cm^2^ for both models. Furthermore, in the MT model, the epithermal neutron intensity at a fast neutron component of 6.8 × 10^−13^ Gy cm^2^ was 41% higher compared with that of 2 × 10^−13^ Gy cm^2^. The *γ*-ray component also caused no significant disadvantages up to several times larger compared with the current recommendation.

## INTRODUCTION

Boron neutron capture therapy (BNCT) is a cancer treatment methodology [1–15], in which a boron agent, such as boronophenylalanine (BPA) or sodium borocaptate, is intravenously administered to the patient to achieve the accumulation of boron in the tumor cells in advance. The patient is then externally irradiated with epithermal neutrons. In BNCT, neutrons are classified into three groups according to their kinetic energy: thermal neutrons below 0.5 eV, epithermal neutrons between 0.5 eV and 10 keV and fast neutrons above 10 keV. Irradiated epithermal neutrons are slowed down to thermal neutrons in the body. The neutrons are captured by the boron elements in the tumor cells, and the tumor cells are killed with high selectivity by α-particles and ^7^Li nuclei with high energy and short ranges generated via the ^10^B(n,α)^7^Li reactions. However, thermal and fast neutrons and coexisting *γ*-rays induce undesired dose components, and their contributions differ according to the beam properties of the neutron source. Several recommendations about these component levels are indicated in the International Atomic Energy Agency (IAEA) publication [16, 17].

A reasonable optimization of the safety of BNCT as well as the development costs of the neutron sources and beam shaping assembly (BSA) for BNCT is crucial for further extending BNCT availability. Acceptable values of each component from the viewpoint of the tolerance doses of normal tissues should be discussed to determine the measures of the beam properties of the neutron sources for BNCT. In a previous study, we developed a simple calculation tool, called SiDE, for determining the dose distribution in a water phantom [18] for easier parameter surveys to derive the measures of the beam properties of the neutron sources for BNCT. SiDE computes the dose distribution in the phantom for sets of arbitrary neutron and *γ*-ray energy spectra. The hydrogen dose, nitrogen dose, other neutron dose, primary and secondary *γ*-ray dose, boron dose, and total dose in tumor, mucosa, skin and other normal tissue are individually computed. It also outputs dose indices, such as peak tumor dose (PTD) and advantage depths (ADs), with a short calculation time of ~1 min.

The purpose of the present study is to evaluate the effect on the dose distribution of changing the beam property from the viewpoints of the skin dose, PTD and ADs. We compute the dose distributions in the phantom using different beam properties obtained by changing the ratio between the thermal and epithermal neutron fluxes (thermal/epithermal ratio), fast neutron component and *γ*-ray component. In the IAEA publication, the current recommendation levels of thermal/epithermal ratio, fast neutron component and *γ*-ray component are 5%, 7 × 10^−13^ and 2 × 10^−13^ Gy cm^2^, respectively. In this study, the tolerance dose of the skin was assumed to be 14 Gy_eq_. The effect on the skin dose is discussed according to this measure, which is nearly equivalent to the threshold dose of dry desquamation (i.e. 14 Gy) [19]. Radiotherapy is typically performed to avoid any treatment-related early serious adverse events, such as Grade 4 or higher. Grade 3 adverse events of the skin tissue are defined as moist desquamation [20] with an 18-Gy threshold dose [19], which is higher than that in the aforementioned assumption. The depths with the dose advantages of the tumor to the normal tissue of 20 Gy_eq_ (AD20) and 25 Gy_eq_ (AD25) are discussed by referring to the prescribed doses of 20 Gy_eq_ in BNCT for advanced salivary gland carcinoma [21] and 18–25 Gy in stereotactic radiosurgery for brain metastases [22].

## METHODS

### Definition of each beam component in the neutron beam

We calculated the dose distributions in a water phantom changing three neutron beam characteristics: thermal/epithermal ratio, fast neutron component and *γ*-ray component in a free-air beam defined in Equations (1)–(3).


(1) \begin{equation*} \mathrm{thermal}/\mathrm{epithermal}\ \mathrm{ratio}\ \left[\hbox{-}\right]=\frac{\mathrm{thermal}\ \mathrm{neutron}\ \mathrm{flux}\ \left[\mathrm{c}{\mathrm{m}}^{-2}\ {\mathrm{s}}^{-1}\right]}{\mathrm{epithermal}\ \mathrm{neutron}\ \mathrm{flux}\ \left[\mathrm{c}{\mathrm{m}}^{-2}\ {\mathrm{s}}^{-1}\right]} \end{equation*}

(2) \begin{equation*} \mathrm{fast}\ \mathrm{neutron}\ \mathrm{component}\ \left[\mathrm{Gy}\ \mathrm{c}{\mathrm{m}}^2\right]=\frac{\mathrm{fast}\ \mathrm{neutron}\ \mathrm{dose}\ \mathrm{rate}\ \left[\mathrm{Gy}\ {\mathrm{s}}^{-1}\right]}{\mathrm{epithermal}\ \mathrm{neutron}\ \mathrm{flux}\ \left[\mathrm{c}{\mathrm{m}}^{-2}\ {\mathrm{s}}^{-1}\right]} \end{equation*}

(3) \begin{equation*} \mathrm{\gamma} -\mathrm{ray}\ \mathrm{c}\mathrm{omponent}\ \left[\mathrm{Gy}\ \mathrm{c}{\mathrm{m}}^2\right]=\frac{\mathrm{\gamma} -\mathrm{ray}\ \mathrm{dose}\ \mathrm{rate}\ \left[\mathrm{Gy}\ {\mathrm{s}}^{-1}\right]}{\mathrm{epithermal}\ \mathrm{neutron}\ \mathrm{flux}\ \left[\mathrm{c}{\mathrm{m}}^{-2}\ {\mathrm{s}}^{-1}\right]} \end{equation*}

### Virtual BSA system and radiobiological coefficients

**Fig. 1:**
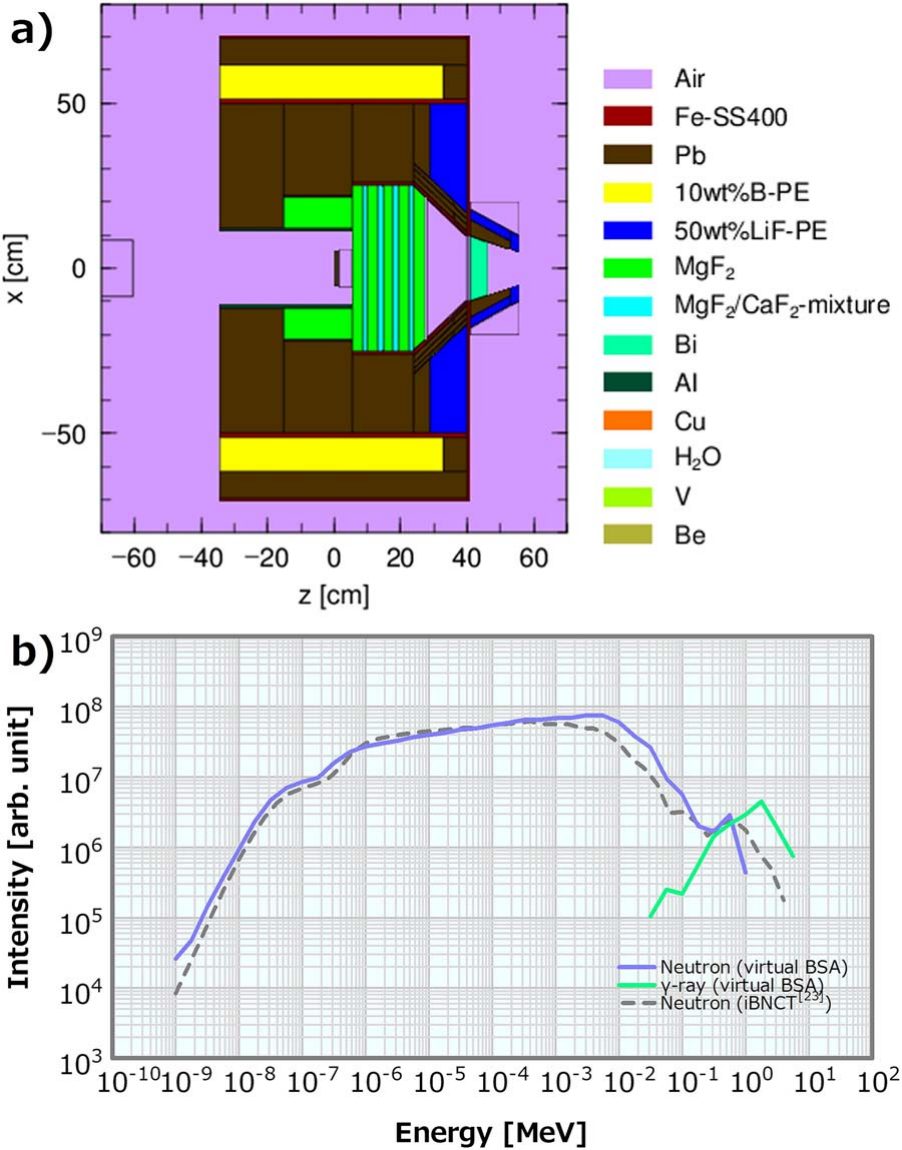
(**a**) A schematic diagram of the virtual BSA system and (**b**) the reference neutron and *γ*-ray energy spectra at the irradiation port of the virtual BSA system and neutron energy spectrum of iBNCT [23].

To evaluate the effects of the changes in the thermal/epithermal ratio, fast neutron component and *γ*-ray component on dose distribution, we used neutron and *γ*-ray energy spectra with the various values of these parameters as the SiDE input. As a reference for these modulated input energy spectra, a pair of reference neutron and *γ*-ray energy spectra was generated using a virtual BSA system with an MgF_2_-based moderator as shown in Fig. 1a. Herein, we assumed a solid lithium target and an accelerated proton energy of 2.8 MeV. Figure 1b shows the reference energy spectra of neutron and *γ*-ray. The neutron energy spectrum obtained from the accelerator-based neutron source at the University of Tsukuba (iBNCT) [23] was also plotted for comparison. Both or either of the reference energy spectra of the virtual BSA was partially modulated by the manners introduced in the subsequent sections. These modulated energy spectra were input to SiDE to calculate the dose distributions in the water phantom for the neutron sources with various beam properties. The beam properties of the reference energy spectra were listed in Table 1, and similar to those of the neutron sources for BNCT at other facilities [23–30].

**Table 1:** Beam properties of the reference neutron and *γ*-ray energy spectra

Parameter	Present value	Recommendations [16]
Thermal/epithermal ratio [−]	5.4%	<5%
Fast neutron component [Gy cm^2^]	5.4 × 10^−13^	<7 × 10^−13^
*γ* ray component [Gy cm^2^]	1.3 × 10^−13^	<2 × 10^−13^

Herein, we assumed a boron agent, i.e. BPA; thus, boron was distributed both in the tumor cells and normal tissue. In the dose calculation using SiDE, the boron concentration was assumed to be 25 ppm in normal cells. The ratio of the boron concentration between the tumor and normal cells was 3.5. The compound biological effectiveness (CBE) of BPA was 4.0 for tumor, 4.9 for mucosa, 2.5 for skin and 1.34 for normal tissue excluding the mucosa and skin (other normal tissue). The relative biological effectiveness was 1.0, 2.9 and 2.4 for *γ*-rays, thermal neutrons and fast neutrons, respectively [30]. The dose indices, namely, ADs, PTD and skin dose, were derived from the dose distributions normalized as the maximum dose in the other normal tissue of 12 Gy_eq_. The skin is defined as the surface region of the phantom within a 2 mm depth. The skin dose was calculated in the region with a boron concentration of 25 ppm and a CBE of 2.5. Although the actual skin tissue is thinner than 2 mm, the 2 mm depth in the simulation calculation is reasonable, considering the pixel size on a treatment planning computed tomography image and the spatial resolution of the contours in the RT Structure. However, this possibly results in an overestimation of the skin dose. Therefore, it should be noted that the overestimated skin dose will cause overly conservative safety.

### Method for varying the thermal/epithermal ratio

**Fig. 2:**
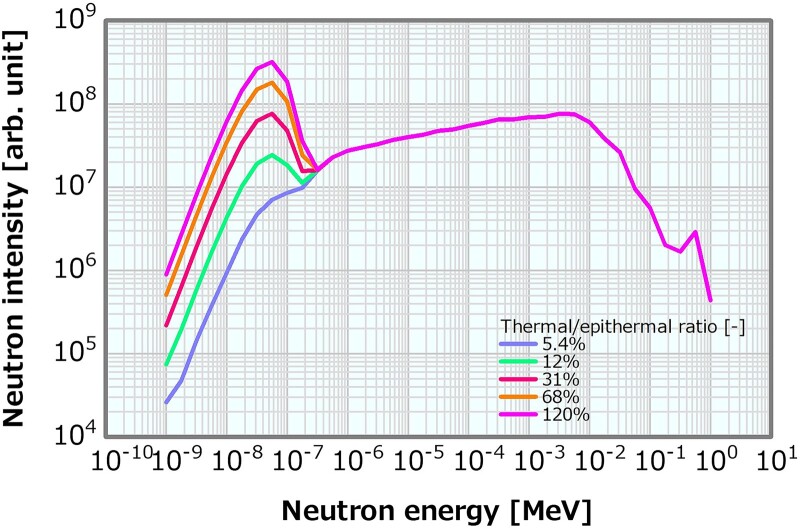
Examples of the neutron energy spectra with different thermal/epithermal ratios.

The Maxwellian distribution Φ(E) = αE^2^exp(−E/k_B_T) was added to the thermal neutron region (<0.5 eV) of the reference neutron energy spectrum shown in Fig. 1b (where Φ denotes the neutron flux; E denotes the neutron energy; k_B_ denotes the Boltzmann coefficient; T denotes the 295-K temperature; and α denotes the intensity coefficient) to generate the neutron energy spectra with different thermal/epithermal ratios. Here α was varied in 19 patterns of 0 and 1 × 10^10^–9 × 10^11^, which corresponded to the thermal/epithermal ratios of 5.4, 6.8, 8.0, 9.2, 11, 12, 13, 15, 16, 17, 19, 31, 43, 56, 68, 81, 92, 100 and 120%. Figure 2 presents examples of the generated neutron energy spectra with different thermal/epithermal ratios. The reference *γ*-ray energy spectrum in Fig. 1b was consistently used as the input *γ*-ray energy spectra in all the calculations to change this ratio.

The skin dose and ADs were derived from the dose distributions. The correlation with the thermal/epithermal ratio was then verified. The thermal/epithermal ratio that violated the skin dose limit of 14 Gy_eq_ was estimated from the results.

### Method for varying the fast neutron component

Two types of models for generating the input energy spectra with varying fast neutron components will be introduced. The first model is called the linear model, in which the distribution in the fast neutron region (>10 keV) in the reference neutron energy spectrum shown in Fig. 1b is replaced by the distribution assuming that the neutron intensity decreases log-linearly with the increasing neutron energy. The maximum neutron energy to be modulated in the intensity is 10 MeV. In this model, the neutron energy spectra with arbitrary fast neutron components can be easily generated by determining the gradient in the fast neutron region. Only the effect of the change in the fast neutron component was observed. The second model is called the moderator thickness (MT) model, which is a more realistic model for the neutron source. The MT model generates energy spectra with different fast neutron components by modifying the MT in the BSA in several patterns (Fig. S1). In this model, the reference energy spectra in Fig. 1b were not used. The neutron and the *γ*-ray energy spectra at each irradiation port of the modified BSAs were utilized as the SiDE input. The first model is considerably easier to use than the second one in terms of producing different fast neutron component data. Therefore, for similar studies in the future, examining the applicability of the first model by comparing their results is important.

In both cases, the dose distributions were computed for a wide range of the fast neutron components increasing from ~2 × 10^−13^ Gy cm^2^, which was the previous recommendation value [17]. The skin dose, PTD and ADs were derived from the dose distributions. The correlation with the fast neutron component was then verified.

#### Fast neutron component varied using the linear model

**Fig. 3:**
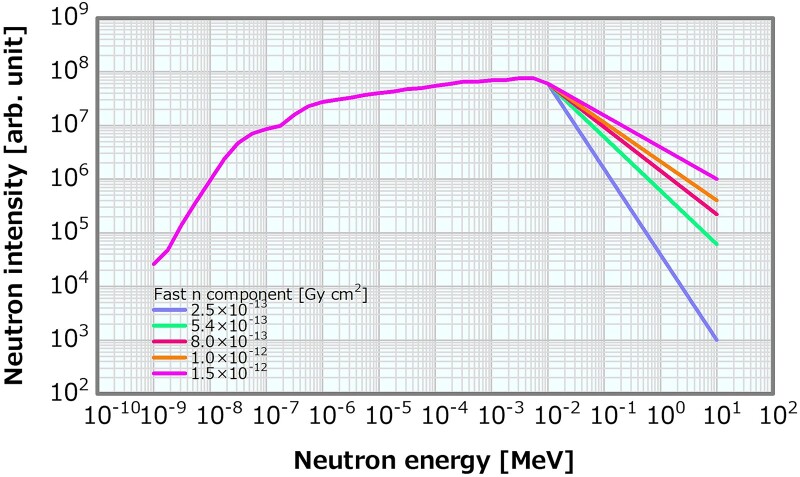
Examples of the neutron energy spectra with the varying fast neutron components generated by the linear model.

The fast neutron region (>10 keV) of the reference neutron energy spectrum presented in Fig. 1b was replaced by seven linear distribution patterns to generate the neutron energy spectra with the fast neutron components of 2.5, 3.5, 5.4, 6.2, 8.0, 10 and 15 × 10^−13^ Gy cm^2^. Figure 3 presents examples of the neutron energy spectra generated by the linear model. As mentioned earlier, the fast neutron flux decreased log-linearly with the increasing neutron energy. The reference *γ*-ray energy spectrum in Fig. 1b was consistently used as the input *γ*-ray energy spectra in all the linear model calculations.

#### Fast neutron component varied by the MT model

**Fig. 4:**
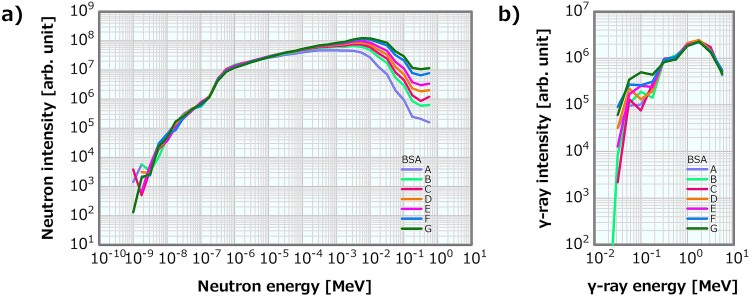
(**a**) Neutron and (**b**) *γ*-ray energy spectra generated by the MT model.

In this model, the MgF_2_ MT in the virtually constructed BSA was modified in seven patterns (i.e. A to G) to generate the neutron energy spectra with the fast neutron components of 2.1, 3.9, 5.1, 6.8, 9.1, 15 and 19 × 10^−13^ Gy cm^2^. The MgF_2_ MT was decreased in the order of A to G. Figure 4 illustrates the neutron and the *γ*-ray energy spectra at the irradiation ports of the BSAs, respectively. Notably, both the fast neutron spectra and the spectra below them were changed. Table 2 lists the beam properties of the energy spectra generated by the MT model. Instead of the reference energy spectra shown in Fig. 1b, pairs of the neutron and the *γ*-ray energy spectra of each BSA system were used as the SiDE input in the MT model to compute the dose distributions.

**Table 2:** BSA and beam properties of the energy spectra generated by the MT model

BSA	MgF_2_ thickness [mm]	Epithermal n flux [× 10^7^ cm^−2^ s^−1^ mA^−1^]	Thermal/epithermal ratio [%]	Fast n component [× 10^−13^ Gy cm^2^]	*γ* ray component [× 10^−13^ Gy cm^2^]
A	285	3.80	1.5	2.1	1.3
B	240	4.68	1.1	3.9	1.0
C	221	5.01	1.0	5.1	0.99
D	202	5.34	0.96	6.8	0.93
E	183	5.59	0.84	9.1	0.85
F	152	5.86	0.83	15	0.81
G	137	5.93	0.77	19	0.77

### Method for varying the *γ*-ray component

**Fig. 5:**
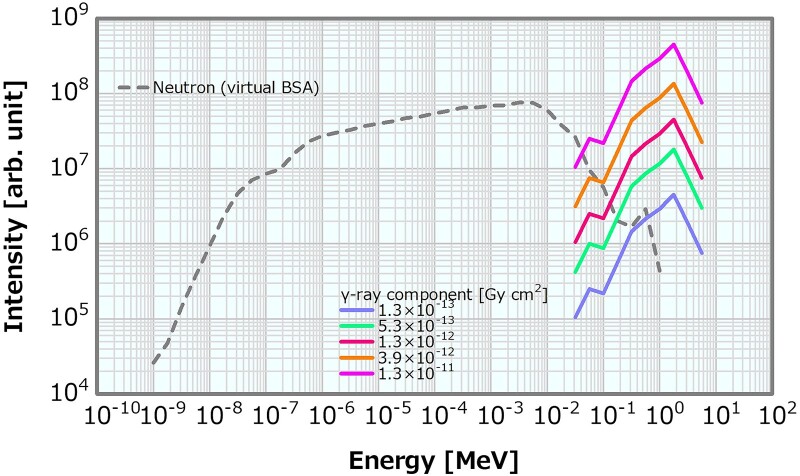
Examples of the neutron and *γ*-ray energy spectra with varying *γ*-ray components.

To calculate the dose distributions for the different *γ*-ray components while using the reference neutron energy spectrum in Fig. 1b as the input neutron energy spectrum, the reference *γ*-ray energy spectrum was multiplied by several integer patterns (Fig. 5) and employed as the input for SiDE. The multipliers were 1, 2, 3, 4, 5, 10, 20, 30, 40, 50 and 100, which corresponded to the *γ*-ray components of 1.3, 2.6, 3.9, 5.3, 6.6 × 10^−13^, 1.3, 2.6, 3.9, 5.3, 6.6 × 10^−12^ and 1.3 × 10^−11^ Gy cm^2^, respectively.

### Method for varying the thermal/epithermal ratio in the case of high and low fast neutron components

**Fig. 6:**
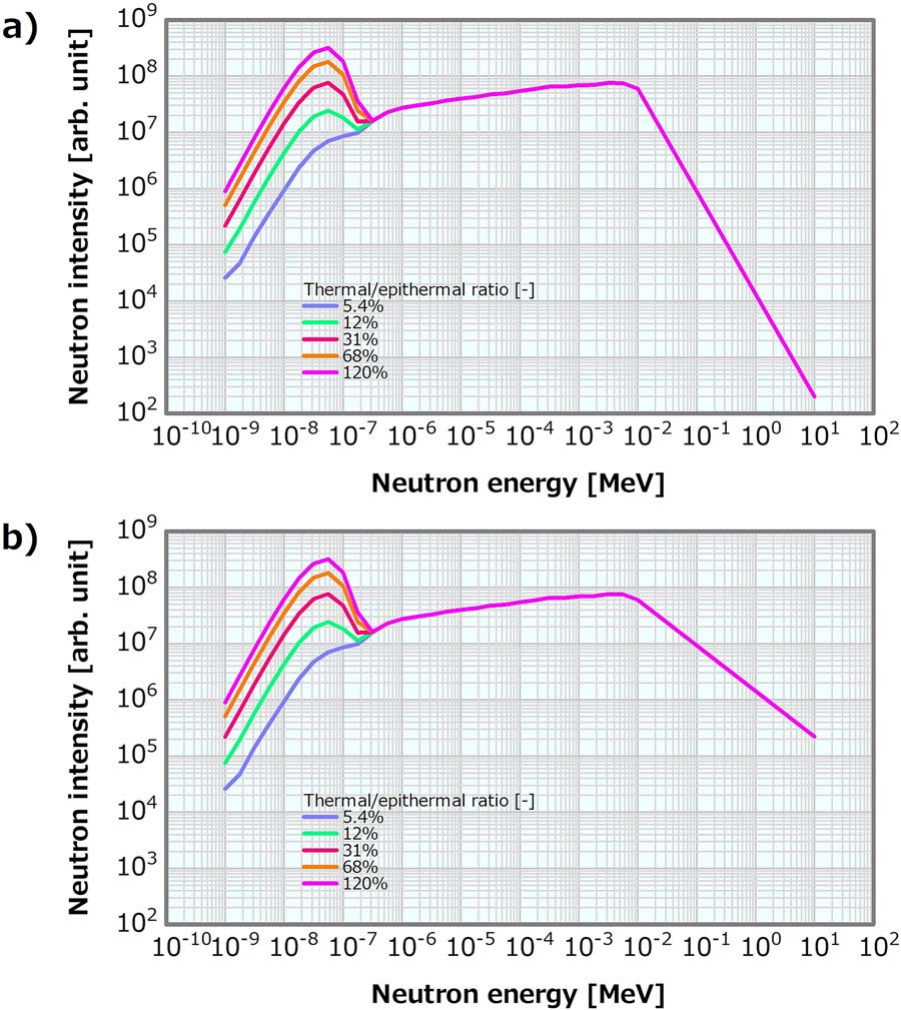
Examples of the neutron energy spectra, in which the thermal/epithermal ratio was varied from 5.4 to 120% in the case of the fast neutron component fixed as (**a**) 2.0 × 10^−13^ Gy cm^2^ and (**b**) 8.0 × 10^−13^ Gy cm^2^ in the linear model.

To evaluate the combined effect of the thermal/epithermal ratio and the fast neutron component, the thermal/epithermal ratio was varied from 5.4 to 120% following the procedure in Section ‘Method for varying the thermal/epithermal ratio’, and the fast neutron component was fixed to either of the two cases of 2.0 × 10^−13^ or 8.0 × 10^−13^ Gy cm^2^, which corresponded to the previous recommended value of the fast neutron component in IAEA TECDOC-1223 [17] and four times of the previous one. Here, we adopted the linear model for the fast neutron component. Figure 6 shows examples of the neutron energy spectra generated for thermal/epithermal ratios in the range of 5.4–120% in the case of the fast neutron component fixed as 2.0 × 10^−13^ or 8.0 × 10^−13^ Gy cm^2^. Here, the reference *γ*-ray energy spectrum in Fig. 1b was consistently used as the input *γ*-ray energy spectrum.

The skin dose and ADs were derived from the dose distribution. The correlation with the thermal/epithermal ratio was then verified. Furthermore, the thermal/epithermal ratio that violated the 14 Gy_eq_ skin dose limit was estimated from the results.

### Method for varying the *γ*-ray component in the case of high and low fast neutron components

**Fig. 7:**
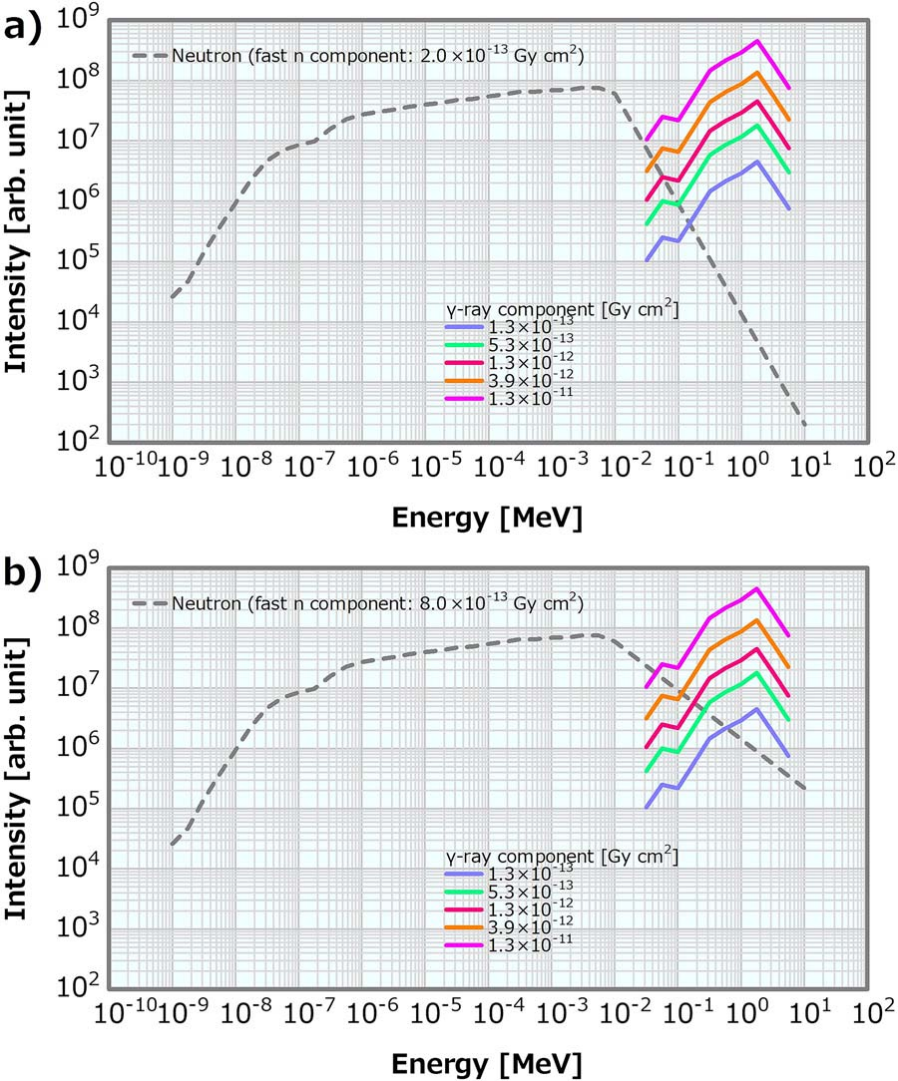
Examples of the neutron and *γ*-ray energy spectra, in which the *γ*-ray component was varied from 1.3 × 10^−13^ to 1.3 × 10^−11^ Gy cm^2^ in the case of the fast neutron component fixed as (**a**) 2.0 × 10^−13^ Gy cm^2^ and (**b**) 8.0 × 10^−13^ Gy cm^2^ in the linear model.

To evaluate the combined effect of the *γ*-ray and fast neutron components, the *γ*-ray component was varied from 1.3 × 10^−13^ to 1.3 × 10^−11^ Gy cm^2^ following the same procedure presented in Section ‘Method for varying the *γ*-ray component’, and the fast neutron component was fixed to either of the two cases of 2.0 × 10^−13^ or 8.0 × 10^−13^ Gy cm^2^ as in 2.6. Figure 7 displays the neutron and *γ*-ray energy spectra generated as the *γ*-ray component from 1.3 × 10^−13^ to 1.3 × 10^−11^ Gy cm^2^ in case of the fast neutron component fixed as 2.0 × 10^−13^ or 8.0 × 10^−13^ Gy cm^2^.

The skin dose, PTD and ADs were derived from the dose distribution. The correlation with the *γ*-ray component was then verified.

## RESULTS

### Thermal/epithermal ratio

**Fig. 8:**
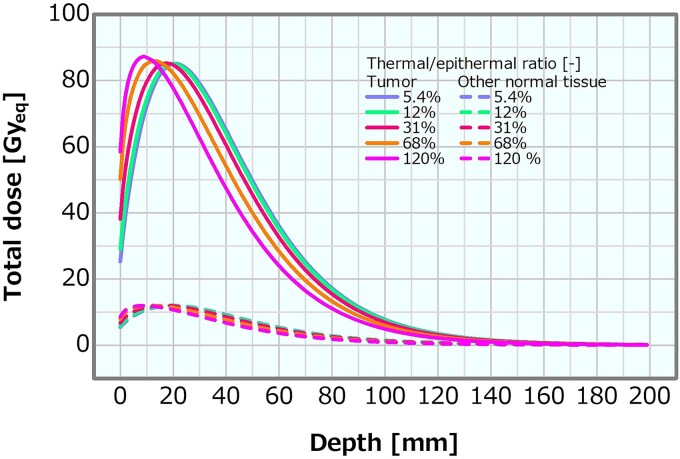
Dose distributions of the tumor and other normal tissues for different thermal/epithermal ratios.

**Fig. 9:**
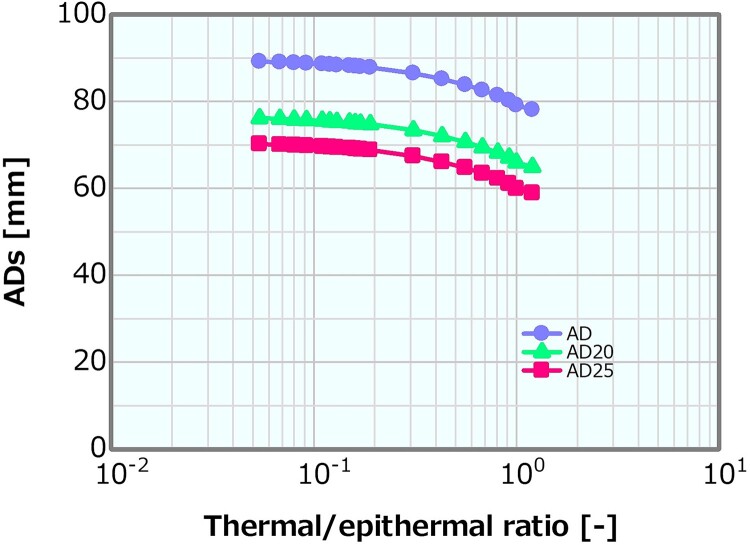
ADs as a function of the thermal/epithermal ratio.

**Fig. 10:**
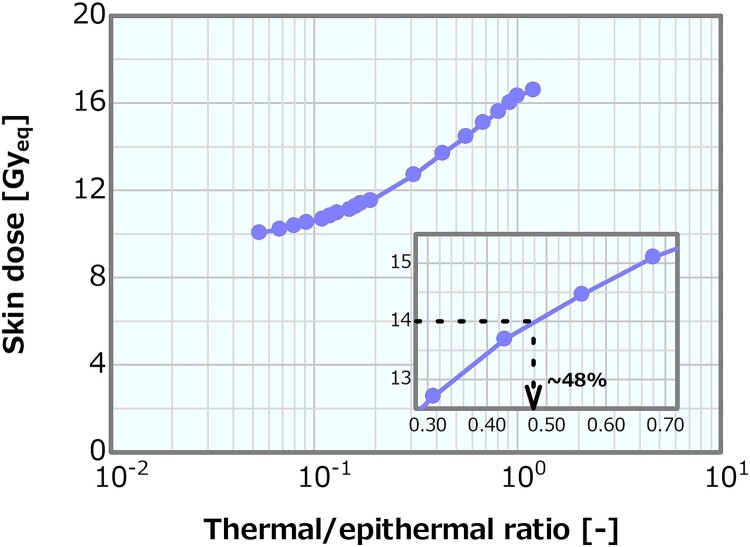
Skin dose as a function of the thermal/epithermal ratio.

Figure 8 shows the dose profiles in the phantom along the central axis of the tumor and other normal tissues for varying thermal/epithermal ratios. A shift in the shallow direction of the overall distribution was observed (i.e. a slight increase in the PTD and a decrease in the PTD depth), particularly for a thermal/epithermal ratio of >31%. The ADs derived from the profiles were plotted as a function of the thermal/epithermal ratio (Fig. 9). They decreased with increasing thermal/epithermal ratio, but the decrements were small (i.e. ~3%), even for the thermal/epithermal ratio of 20–30%. Figure 10 depicts the skin dose as a function of the thermal/epithermal ratio. The skin dose increased with increasing thermal/epithermal ratio and reached a tolerance dose of the skin of 14 Gy_eq_ at a thermal/epithermal ratio of ~48%.

### Fast neutron component

#### Linear model

**Fig. 11:**
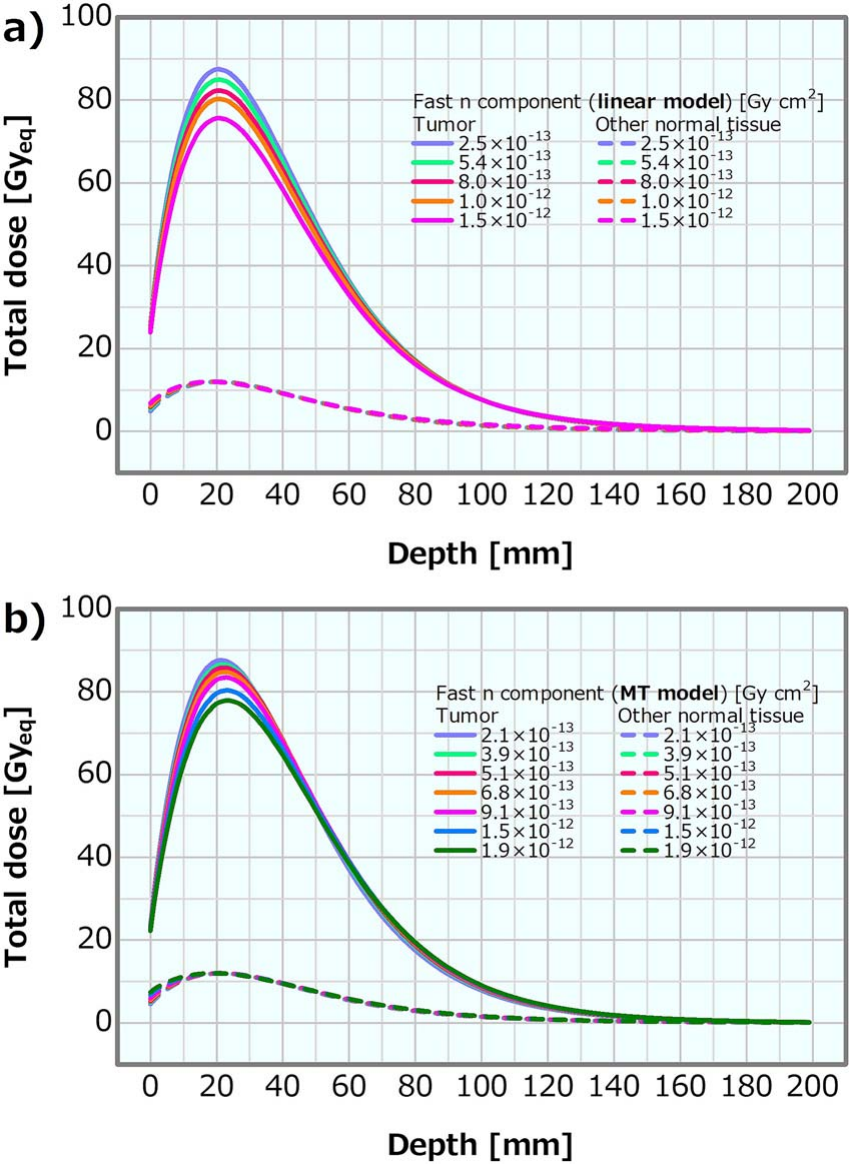
Dose distributions of the tumor and other normal tissues for different fast neutron components varied by the (**a**) linear and (**b**) MT models.

**Fig. 12:**
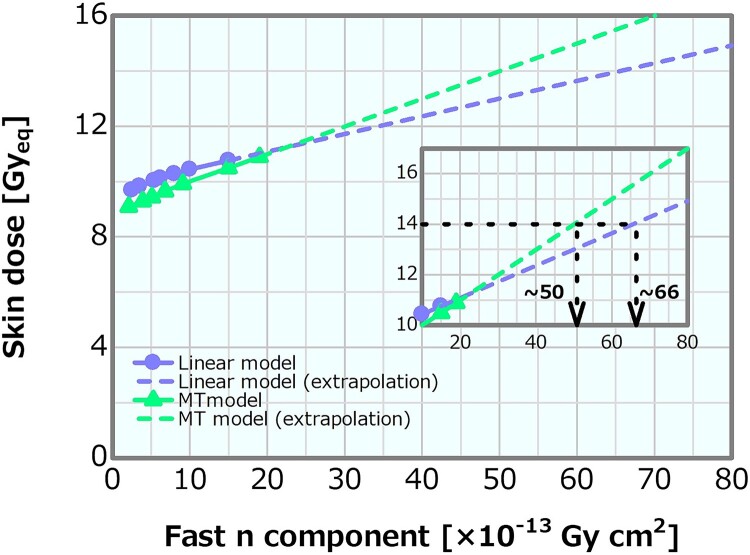
Skin dose as a function of the fast neutron component varied by the linear and MT models. The dashed lines represent the extrapolation results of both models.

**Fig. 13:**
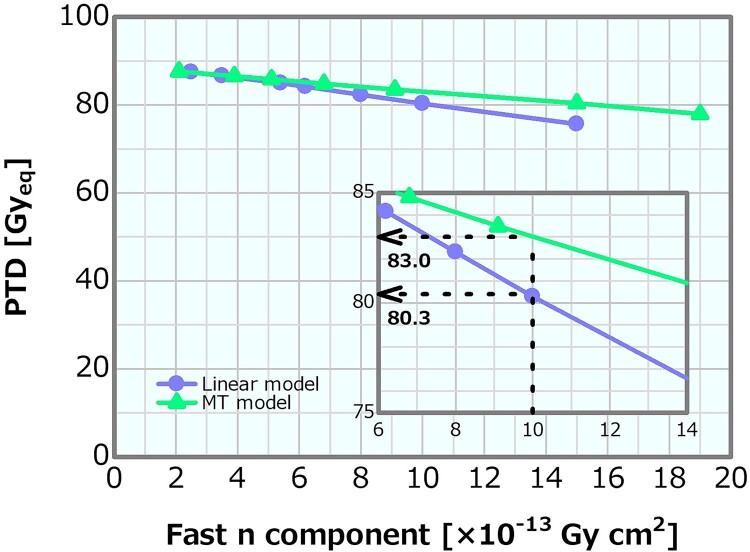
PTD as a function of the fast neutron component varied by the linear and MT models.

**Fig. 14:**
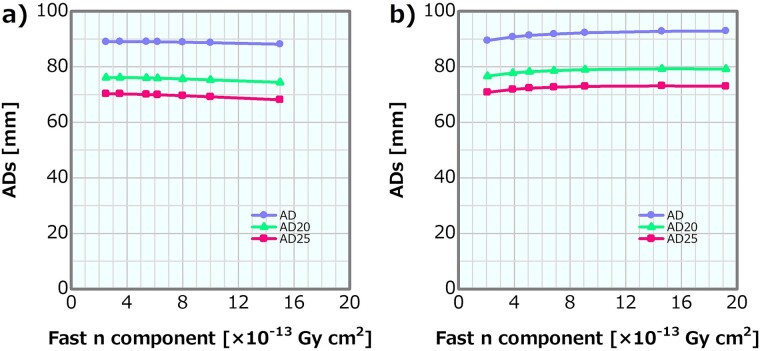
ADs as a function of the fast neutron component in the (**a**) linear and (**b**) MT models.

Figure 11a shows the dose profiles in the phantom along the central axis of the tumor and other normal tissues for the varying fast neutron components in the linear model. The skin dose, PTD and ADs were plotted as a function of the fast neutron component varied by the linear model; the corresponding plots are presented in Figs 12–14a, respectively. The skin dose increased, while the PTD and ADs slightly decreased with the increasing fast neutron component. The skin dose was always <14 Gy_eq_ in all the evaluated cases; hence, the extrapolation line was also plotted in Fig. 12. From the extrapolation result, the skin dose was estimated to reach 14 Gy_eq_ at a fast neutron component of ~6.6 × 10^−12^ Gy cm^2^ for this model.

#### MT model

Figure 11b depicts the dose profiles in the phantom along the central axis of the tumor and other normal tissues for the varying fast neutron components in the MT model. The skin dose, PTD and ADs were also plotted as a function of the fast neutron component in the MT model; the corresponding plots are presented in Figs 12–14b, respectively. The skin dose and the PTD indicated trends similar to those in the linear model. The skin dose was always <14 Gy_eq_ in all the evaluated cases; hence, the extrapolation line was also plotted in Fig. 12. From the extrapolation result in this model, the skin dose was estimated to reach 14 Gy_eq_ at a fast neutron component of ~5.0 × 10^−12^ Gy cm^2^. For this model, the ADs slightly increased with the increasing fast neutron component. Additionally, ~41% of the increment in the epithermal neutron intensity was expected for the fast neutron component of 6.8 × 10^−13^ Gy cm^2^ compared with that of 2.1 × 10^−13^ Gy cm^2^ in the MT model (Table 2).

### γ-ray component

**Fig. 15:**
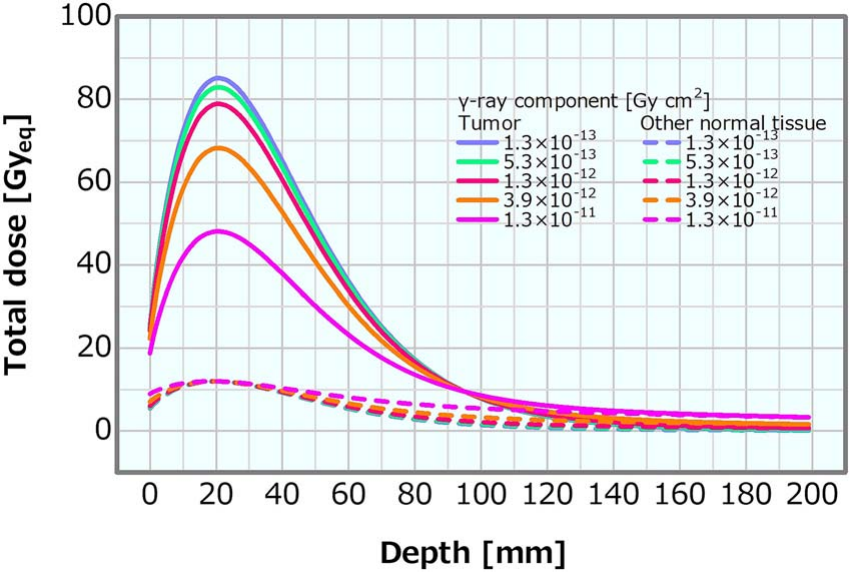
Dose distributions of the tumor and other normal tissues for different *γ*-ray components.

**Fig. 16:**
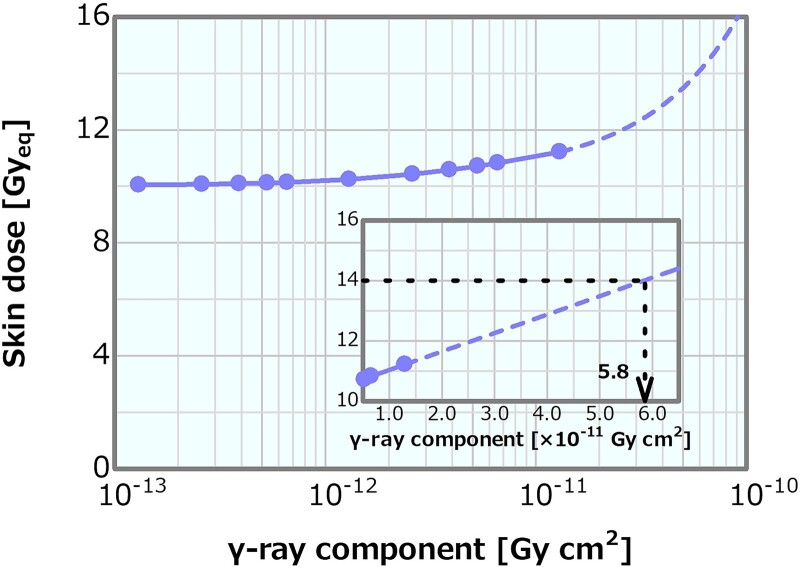
Skin dose as a function of the *γ*-ray component. The dashed line represents the extrapolation result.

**Fig. 17:**
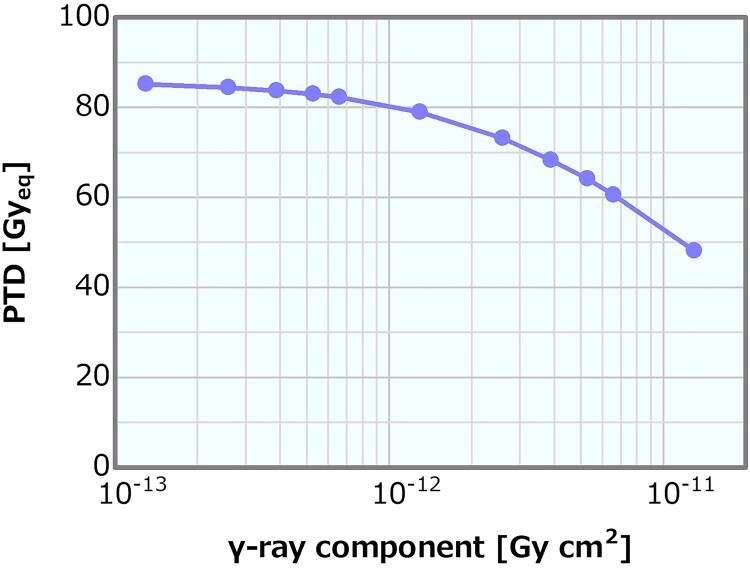
PTD as a function of the *γ*-ray component.

**Fig. 18:**
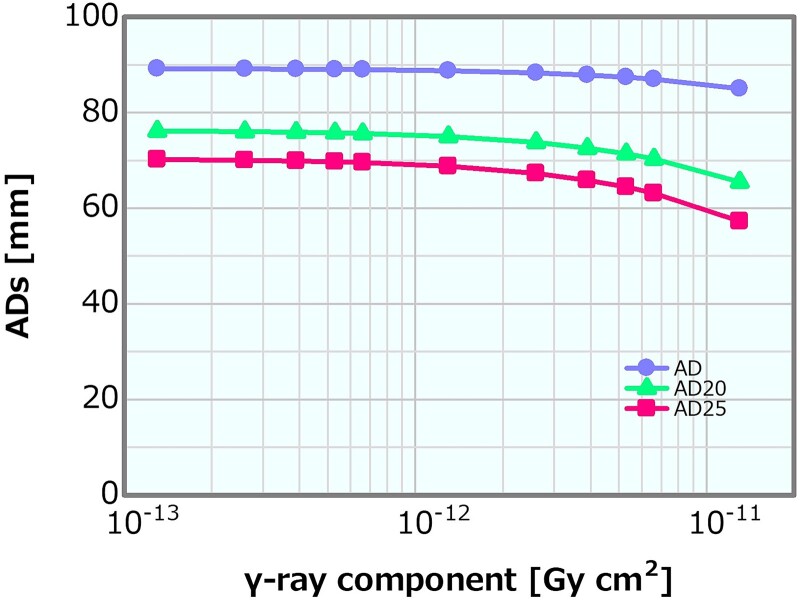
ADs as a function of the *γ*-ray component.

Figure 15 shows the dose profiles in the phantom along the central axis of the tumor and other normal tissues for the varying *γ*-ray component. The skin dose, PTD and ADs were plotted as a function of the *γ*-ray component; the corresponding plots are presented in Figs 16–18, respectively. The skin dose increased, while the PTD and the ADs decreased with the increasing *γ*-ray component. The skin dose was always <14 Gy_eq_ in all the evaluated cases and estimated to reach 14 Gy_eq_ at a *γ*-ray component of ~5.8 × 10^−11^ Gy cm^2^ from the extrapolation result (Fig. 16). Although the effect on the PTD was relatively more severe than that on the skin dose and the ADs, these variations were small up to a *γ*-ray component of ~1 × 10^−12^ Gy cm^2^.

### Thermal/epithermal ratio in the case of the high and low fast neutron components

**Fig. 19:**
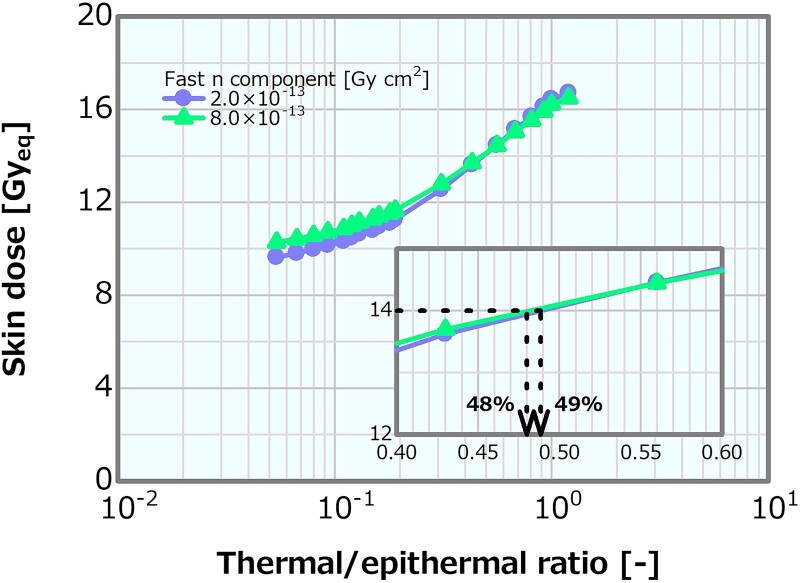
Skin dose as a function of the thermal/epithermal ratio for the fast neutron components of 2 × 10^−13^ and 8 × 10^−13^ Gy cm^2^.

**Fig. 20:**
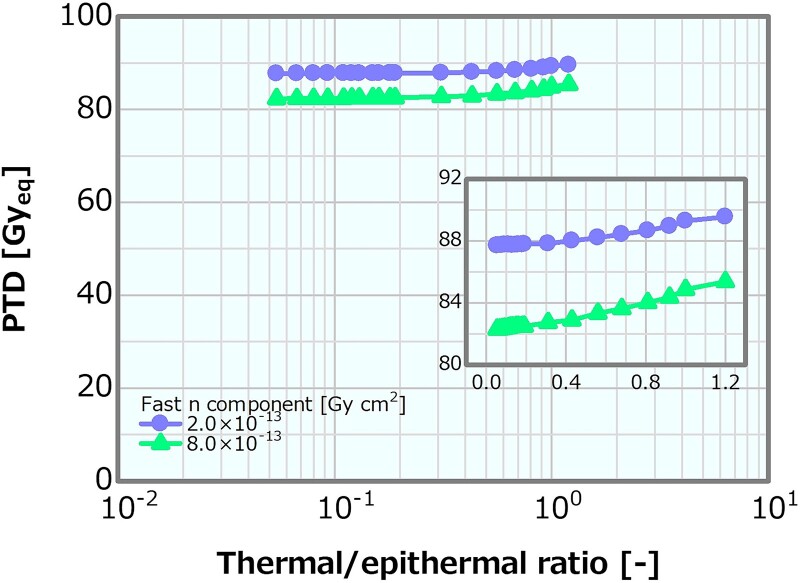
PTDs as a function of the thermal/epithermal ratio for the fast neutron components of 2 × 10^−13^ and 8 × 10^−13^ Gy cm^2^.

Concerning the combination of the fixed fast neutron component of 2 × 10^−13^ or 8 × 10^−13^ Gy cm^2^ and the varying thermal/epithermal ratio, Fig. 19 depicts the skin dose as a function of the thermal/epithermal ratio. The skin dose increased with the increasing thermal/epithermal ratio, reaching a 14-Gy_eq_ tolerance dose at the thermal/epithermal ratios of ~49 and 48% at the fast neutron components of 2 × 10^−13^ and 8 × 10^−13^ Gy cm^2^, respectively. These results were consistent with that discussed in Section ‘Thermal/epithermal ratio’. The ADs also indicated a trend similar to that in Section ‘Thermal/epithermal ratio’ (Fig. S2). Although AD20 and AD25 at the fast neutron component of 2 × 10^−13^ Gy cm^2^ were slightly higher than those at the fast neutron component of 8 × 10^−13^ Gy cm^2^ (i.e. ~0.9% difference at the maximum corresponding to 0.63 mm for AD25), the ADs indicated similar trends in both cases and decreased with the increasing thermal/epithermal ratio. Figure 20 shows the PTD as a function of the thermal/epithermal ratio. The PTD also indicated a trend similar to that discussed in Section ‘Thermal/epithermal ratio’, particularly slightly increasing for a thermal/epithermal ratio of >31%.

### γ-ray component in the case of the high and low fast neutron components

**Fig. 21:**
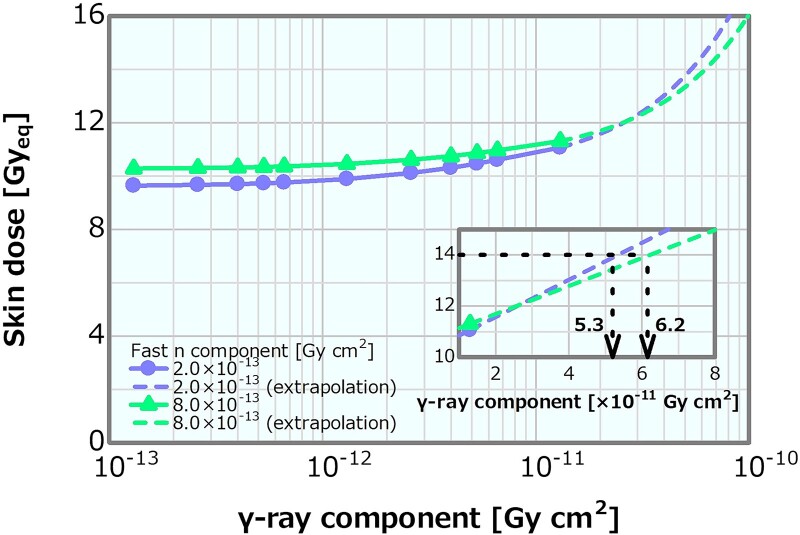
Skin dose as a function of the *γ*-ray component for the fast neutron components of 2 × 10^−13^ and 8 × 10^−13^ Gy cm^2^. The dashed lines represent the extrapolation results.

Concerning the combination of the fixed fast neutron component of 2 × 10^−13^ or 8 × 10^−13^ Gy cm^2^ and the varying *γ*-ray components, the skin dose is depicted in Fig. 21 as a function of the *γ*-ray component. The skin dose increased with the increasing *γ*-ray component, reaching a 14-Gy_eq_ tolerance dose at the *γ*-ray components of ~5.3 × 10^−11^ and 6.2 × 10^−11^ Gy cm^2^ at the fast neutron components of 2 × 10^−13^ and 8 × 10^−13^ Gy cm^2^, respectively. These results were almost consistent to that presented in Section ‘*γ*-ray component’. The ADs and PTD also indicated similar trends as discussed in Section ‘*γ*-ray component’. The ADs indicated similar trends in both cases and decreased with the increasing *γ*-ray component (Fig. S3). The PTD at the fast neutron component of 2 × 10^−13^ Gy cm^2^ was always slightly higher than that at the fast neutron component of 8 × 10^−13^ Gy cm^2^ (Fig. S4). Consequently, no severe effect on the skin dose, ADs and PTD other than that described in Section ‘*γ*-ray component’ was observed. Thus, although the dose advantages of the tumor decreased with the increasing *γ*-ray component, no severe effect was found for the *γ*-ray component of <1 × 10^−12^ Gy cm^2^.

## DISCUSSIONS

For the thermal/epithermal ratios of ~25%, the decrement in AD25 was 3%, and for 48%, the skin dose reached 14 Gy_eq_. Notably, even for the thermal/epithermal ratio that was five times higher than the current recommended value of 5%, the decrement in AD25 was small and the skin dose variation was acceptable. For the thermal/epithermal ratio of 10%, which was double the current recommendation, the skin dose, PTD and AD25 were 10.6 Gy_eq_, 85.1 Gy_eq_ and 69.7 mm, respectively. Hence, from the viewpoints of the skin dose and the ADs, a thermal/epithermal ratio that was a few times higher than the current recommendation was considered not to cause a severe effect on the dose distribution.

In the linear model, the skin dose increment and the PTD decrement were ~10% between the fast neutron components of 1.35 × 10^−12^ and 1.17 × 10^−12^ Gy cm^2^. The skin dose, PTD and AD25 for the fast neutron component of 1 × 10^−12^ Gy cm^2^, which was ~43% larger than the current recommended value of 7 × 10^−13^ Gy cm^2^, were 10.4 Gy_eq_, 80.3 Gy_eq_ and 69.2 mm, respectively.

Meanwhile, in the MT model, the skin dose increment and the PTD decrement were 10% between the fast neutron components of 1.00 × 10^−12^ and 1.76 × 10^−12^ Gy cm^2^. The skin dose, PTD and AD25 were ~10.0, 83.0 Gy_eq_ and 73.0 mm, respectively, for the fast neutron component of 1 × 10^−12^Gy cm^2^. The current recommendation value of the fast neutron component is 7 × 10^−13^ Gy cm^2^. If the value is increased to 1 × 10^−12^ Gy cm^2^, there is no severe effect on the dose distribution from the viewpoint of the skin dose, PTD and ADs. Furthermore, the slight increase in the ADs in this model was attributed to the changes in the energy spectra according to the increase in the fast neutron component (Table 2). A fast neutron component of 6.8 × 10^−13^ Gy cm^2^ was expected to achieve a 41% increase in the epithermal neutron intensity compared with 2.1 × 10^−13^ Gy cm^2^. This means that, notably, an extraordinary restriction on the fast neutron component may cause a reduction in the epithermal neutron yield. Thus, the update of the recommendation value of the fast neutron component in the current recommendation can be concluded to be safe and reasonable.

Both the linear and MT models indicated similar skin dose and PTD trends. Although the increasing effect in the ADs in the MT model was not reproduced, the difference in ADs in both methods is small. Therefore, the linear model is a simple and reasonable method for generating the neutron energy spectra modulated in the fast neutron components.

An increase in the *γ*-ray component can mainly cause a decrease in the PTD; however, the impact was not considered severe for the *γ*-ray component of <1 × 10^−12^ Gy cm^2^. The effects on the skin dose and ADs were small compared with those on the PTD. Moreover, the *γ*-ray component indicating 14 Gy_eq_ of the skin dose was estimated as extremely high (i.e. >10^−11^ Gy cm^2^). The PTD and AD25 decrements and the skin dose increment were 3% at the *γ*-ray components of ~5.3 × 10^−13^, 2.0 × 10^−12^ and 2.0 × 10^−12^ Gy cm^2^, respectively, compared with those at 2.0 × 10^−13^ Gy cm^2^. A *γ*-ray component that was a few times larger than the current recommendation was considered not to cause any severe effect. The skin dose, PTD and AD25 for the *γ*-ray component of 5 × 10^−13^ Gy cm^2^, which was 2.5 times larger than the current recommended value of 2 × 10^−13^ Gy cm^2^, were 10.1 Gy_eq_, 83.1 Gy_eq_ and 69.7 mm, respectively.

For all the combinations of the fast neutron component fixed to a value around the previous recommendation or four times higher and the thermal/epithermal ratio or *γ*-ray component, the obtained results indicated trends similar to those obtained from the single-value variation. Therefore, from the viewpoints of the skin dose, PTD and ADs, even if the thermal/epithermal ratio or *γ*-ray component with a value of a few times higher than the current recommendation and the fast neutron component equivalent to the current recommendation are simultaneously assumed, the combined effects will not cause any severe effect.

In the SiDE calculation, a parallel beam was assumed for both the neutron and *γ*-ray. IAEA publication [16] defines neutron beam divergence as the ratio between the total neutron current and the total neutron flux (C/F), with a recommended value >0.7. We will discuss the effect of the beam divergence on the obtained results by comparing the dose distributions in the case of 0.729 C/F with the aforementioned results. The calculation at the 0.729 C/F was performed using the Monte Carlo simulation code, Particle and Heavy Ion Transport Code System (PHITS) [31]; the results are presented in detail in the Supplementary Material. The PHITS results indicated a skin dose increment of ~17.6%, a PTD decrement of 0.935% and an AD25 increment of 2.17% in comparison with the SiDE results (Table S1). The beam divergence only exhibited a slight effect on AD25 and PTD and was within the statistical errors. Conversely, the effect on the skin dose was not negligible. However, even if a large increase caused by the beam divergence was assumed, the skin doses were kept <14 Gy_eq_ at a few times larger values of the *γ*-ray component and thermal neutron ratio compared with the recommended values in the IAEA publication. Thus, the recommendation values of the thermal/epithermal ratio and *γ*-ray component are also set as clinically safe values.

## CONCLUSIONS

In this paper, we calculated the dose distributions in the water phantom for a series of energy spectra with various beam properties using the developed simple dose calculation tool, called SiDE, to evaluate the effect of the differences in the beam properties of the neutron source for BNCT. The verification results of the thermal/epithermal ratio revealed that the ratio violating the tolerance dose level of the skin (14 Gy_eq_) and decreasing the ADs by 3% was >20%. The effect of the differences in the fast neutron component was verified using two different models, namely the linear and MT models. Their results indicated that the skin dose was nearly 10 Gy_eq_, even when considering a fast neutron component of 1 × 10^−12^ Gy cm^2^. In the MT model, an increase in the epithermal neutron intensity caused by the reduction of the MT in the BSA was expected to be ~40% when considering a fast neutron component that is a few times higher than the current recommendation. The changes in the dose distribution based on the differences in the *γ*-ray component were small for the *γ*-ray component <1 × 10^−12^ Gy cm^2^. Notably, in all the evaluated cases of the fast neutron and *γ*-ray components, the skin dose was always <14 Gy_eq_. The obtained results might not be rigorous because SiDE calculated the doses based on parallel-beam simplification. However, the trends found in this work will almost remain unchanged because beam parallelism is usually good, even in the actual BSA. Therefore, even in an actual beam, the mitigation of the recommended values will cause only small changes in the physical properties of the dose distribution in a phantom. Thus, the effectiveness and safety of the BNCT treatment will remain almost unchanged even after mitigation of the recommended values.

## Supplementary Material

zzz_supplementary_ver240924_clean_unblinded
