# Supplementary material for: Effect of neutron beam properties on dose distributions in a water phantom for boron neutron capture therapy
Source: J Radiat Res. 2024 Oct 4;65(6):765–75. doi: 10.1093/jrr/rrae076 (PMC11630089; doi:10.1093/jrr/rrae076)
Supplement: zzz_supplementary_ver240924_clean_unblinded [file zzz_supplementary_ver240924_clean_unblinded.docx]

**Supplementary Material**

Manuscript for *Journal of Radiation Research*

Article type: Regular paper

Title:

Effect of neutron beam properties on dose distributions in a water phantom for boron neutron capture therapy

Short title:

Effect of neutron beam properties for BNCT

Authors:

Akihisa Ishikawa^1,2,*^, Hiroki Tanaka^3^, Satoshi Nakamura^4^, Hiroaki Kumada^5^, Yoshinori Sakurai^3^, Kenichi Watanabe^6^, Sachiko Yoshihashi^2^, Yuki Tanagami^2^, Akira Uritani^2^, and Yoshiaki Kiyanagi^7^

Institutional affiliations:

^1^ Nuclear Science and Engineering Center, Japan Atomic Energy Agency, 2-4 Shirakata, Tokai-mura, Ibaraki 319-1195, Japan

^2^ Graduate School of Engineering, Nagoya University, Furo-cho, Chikusa-ku, Nagoya, Aichi 464-8603, Japan

^3^ Integrated Radiation and Nuclear Science, Kyoto University, 2, Asashiro-Nishi, Kumatori-cho, Sennan-gun, Osaka 590-0494, Japan

^4^ Division of Radiation Safety and Quality Assurance, National Cancer Center Hospital, 5-1-1, Tsukiji, Chuo-ku, Tokyo 104-0045, Japan

^5^ Faculty of Medicine, University of Tsukuba, Tsukuba, Ibaraki 305-8575, Japan

^6^ Graduate School of Engineering, Kyushu University, 744, Motooka, Nishi-ku, Fukuoka 819-0395, Japan

^7^ Hokkaido University, Sapporo, Hokkaido 060-8628, Japan

Corresponding author’s information:

Name: Akihisa Ishikawa

Address: 2-4 Shirakata, Tokai-mura, Ibaraki 319-1195, Japan

TEL: +81-29-282-5441, E-mail: [ishikawa.akihisa@jaea.go.jp](mailto:ishikawa.akihisa@jaea.go.jp)

Supplementary File:

**S1. MT model**

To generate the input energy spectra with varying fast neutron components, the MT model was used in the present study. In this model, the moderator thickness in the BSA was adjusted in several patterns to change the fast neutron component.

Seven patterns of the virtual BSA with different MT as shown in Fig. S1 were simulated. The MgF_2_ MT was decreased in the order of A to G. The neutron and γ-ray energy spectra of each BSA were computed and input to SiDE.


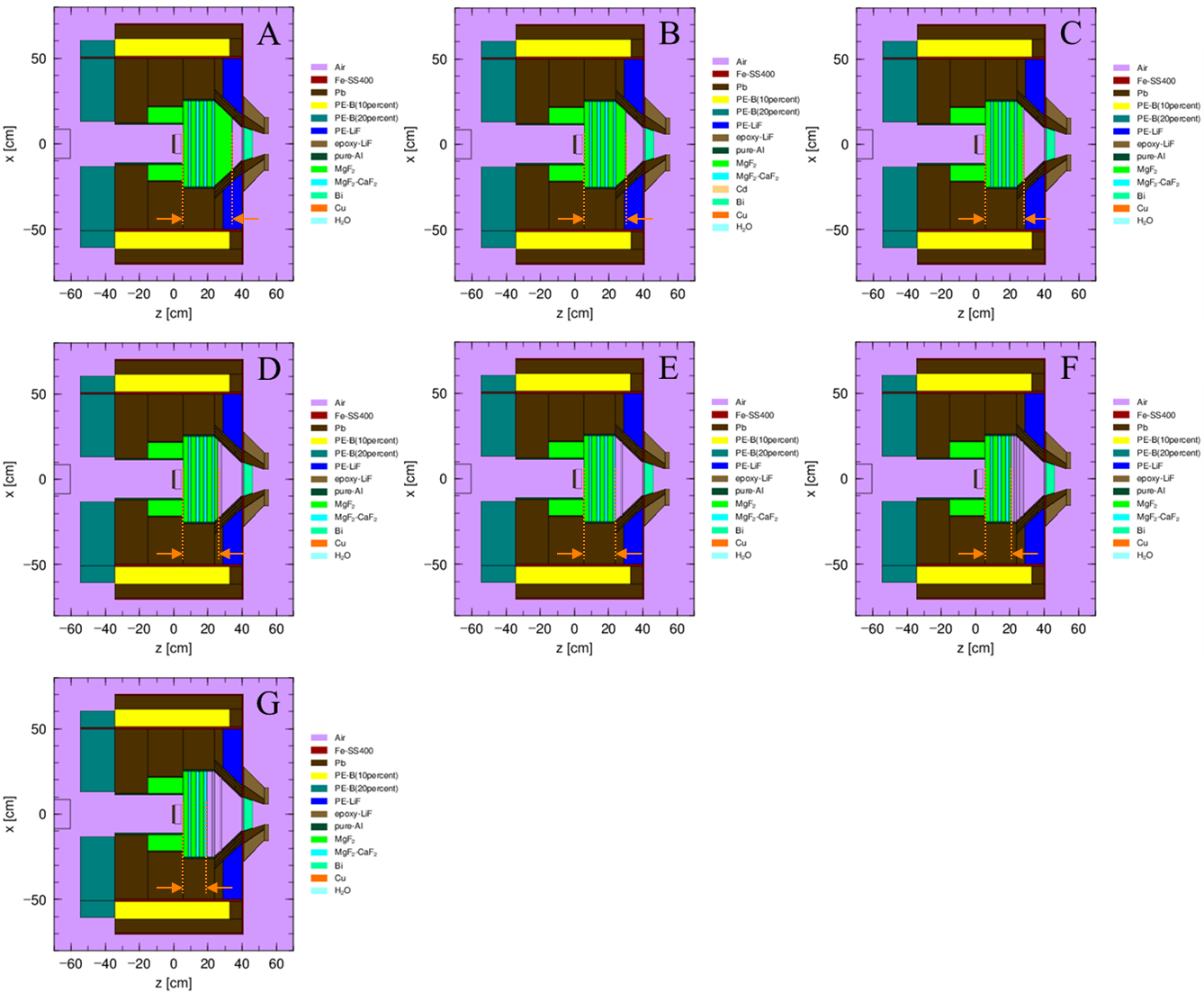


Fig. S1. Schematic diagrams of virtual BSA systems in MT model

**S2. ADs and PTDs at the high and low fast neutron components**

***S2.1. ADs as a function of the thermal/epithermal ratio at the high and low fast neutron components***


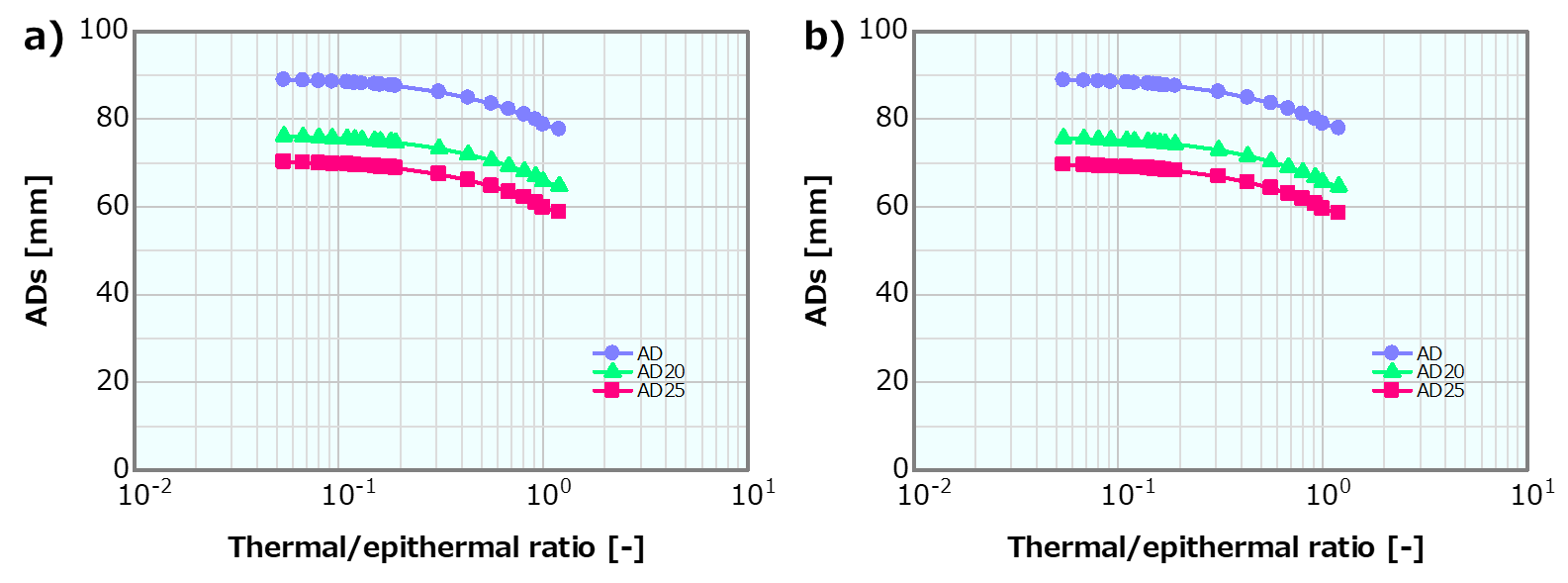


Fig. S2. Advantage depths (ADs) as a function of the thermal/epithermal ratio at the fast neutron components of a) 2 × 10^−13^ and b) 8 × 10^−13^ Gy cm^2^

***S2.2. ADs and PTDs as a function of the γ-ray ratio at the high and low fast neutron components***


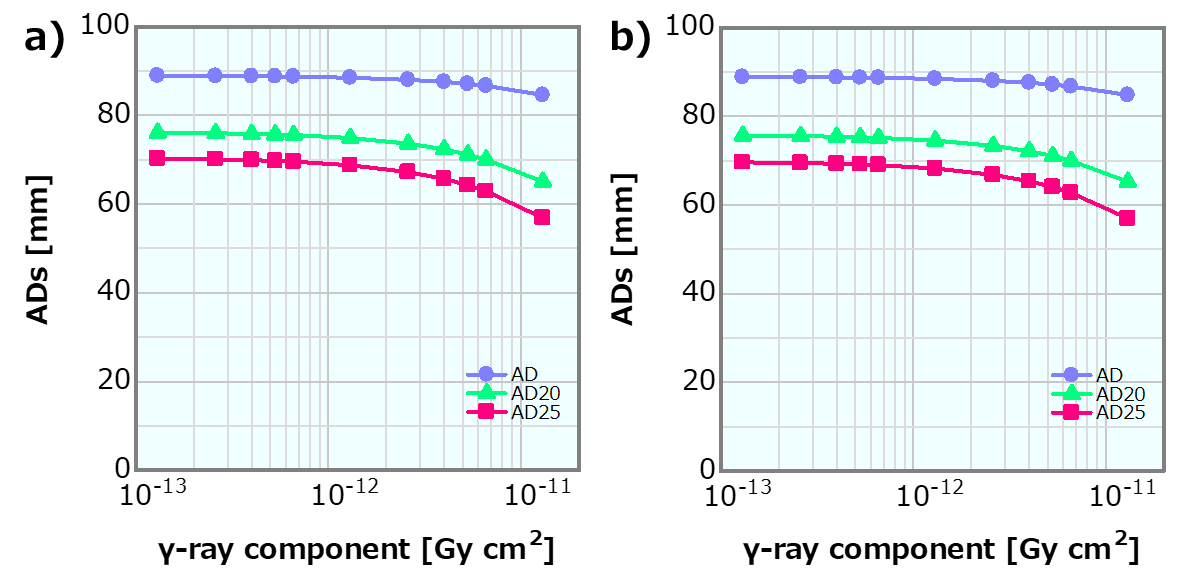


Fig. S3. Advantage depths (ADs) as a function of the γ-ray component at the fast neutron components of a) 2 × 10^−13^ and b) 8 × 10^−13^ Gy cm^2^


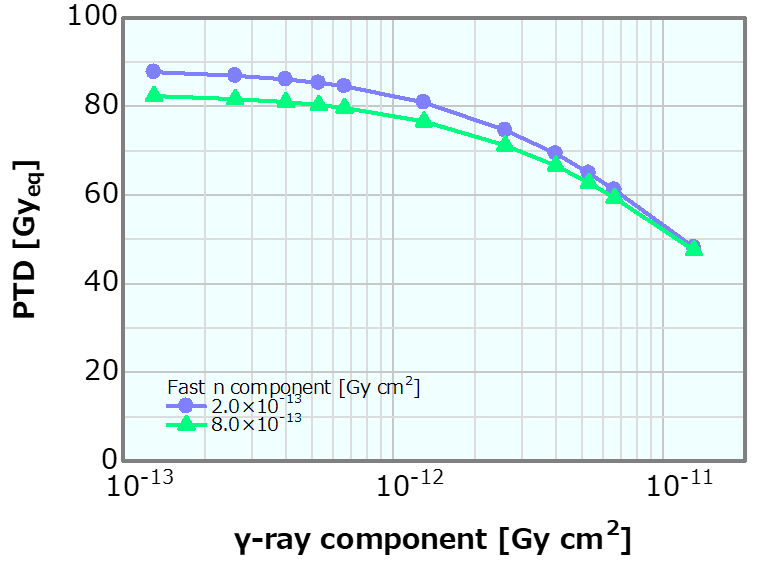


Fig. S4. PTDs as a function of the γ-ray component for the fast neutron components of 2 × 10^−13^ and 8 × 10^−13^ Gy cm^2^

**S3. Effect of the beam divergence**

***S3.1. Introduction***

In the present study, the SiDE assumed a parallel beam as a neutron and γ-ray sources, namely, no beam divergence. However, in an actual case, the neutron beam has an angular distribution, which is represented by a measure called the current-flux ratio (C/F). To evaluate the effect of the beam divergence on the dose distribution, we compare the dose distribution results between the SiDE and PHITS from the viewpoint of the skin dose, peak tumor dose (PTD), and advantage depth 25 (AD25).

***S3.2. Methods***

*S3.2.1. Dose calculation by the PHITS*

To calculate dose distribution by the PHITS, a BSA system with the C/F of nearly 0.7 and beam diameter of 10 cm was virtually constructed. The constructed BSA system was shown in Fig. S5. The C/F of the BSA was 0.729. The thermal/epithermal ratio, fast neutron component, and γ-ray component were 1.11%, 2.17 × 10^−13^ Gy cm^2^, and 1.08 × 10^−13^ Gy cm^2^, respectively. A water phantom with a volume of 200 × 200 × 200 mm^3^ was set in front of the beam exit of the BSA. The dose profile along the central axis in the water phantom was calculated by using 200 coin-shaped tallies with the thickness of 1 mm and diameter of 10 mm lined at the depth in the water phantom from 0 to 200 mm. The history number of this calculation was 4 × 10^8^. The calculated dose distribution was normalized by the maximum dose in normal tissue of 12 Gy_eq_. and the skin dose, PTD, and AD25 were derived.


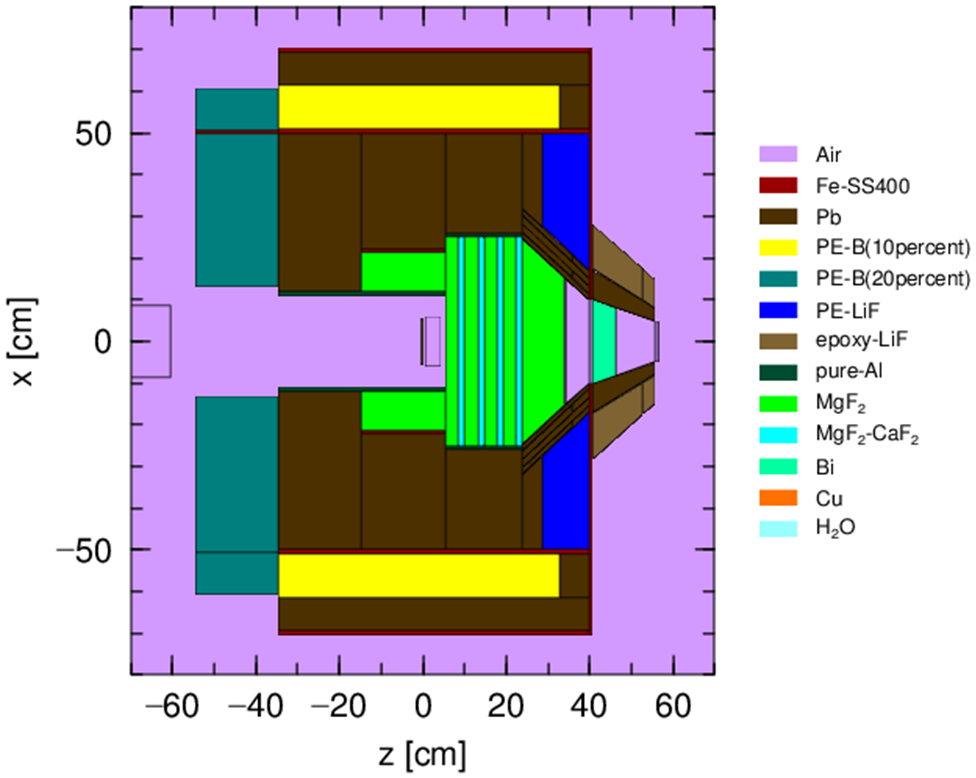


Fig. S5. The virtual BSA system with the C/F of 0.729 and beam diameter of 10 cm.

*S3.2.2. Dose calculation by the SiDE*

The energy spectra of neutron and γ-ray used in the SiDE calculation were those obtained at the beam exit of the virtual BSA by the PHITS calculation. Figure S6 depicts the energy spectra of neutron and γ-ray at the beam exit. The energy spectra were input to the SiDE, which calculates the dose distribution under the condition of the parallel beam. The calculated dose distribution was normalized by maximum dose in normal tissue of 12 Gy_eq_. and the skin dose, PTD, and AD25 were derived.

Fig. S6. The energy spectra of neutron and γ-ray at the beam exit of the virtual BSA shown in Fig. S5

***S2.3. Results and Discussions***

Figure S7 depicts the normalized dose distribution in the water phantom of normal tissue dose and tumor dose calculated by the PHITS and SiDE. The tumor dose by the PHITS was higher than that by the SiDE in the shallow region (from 0 to 10 mm) and deep region (>80 mm). The PTD and AD25 were derived from the distribution, and listed in Table S1. The skin dose at the depth of 2 mm in the water phantom was also listed. The skin dose and AD25 increased about 17.6% and 2.17 %, respectively, and PTD decreased about 0.935% due to the beam divergency with the C/F of 0.729. The effect of parallel beam appeared mainly in skin dose but the effect is not so large, and the differences in AD25 and PTD were very small, which were within statistical errors.

Fig. S7. The normalized dose distribution of normal tissue dose and tumor dose calculated by the PHITS and SiDE. The ratio of the tumor dose by the PHITS to that by the SiDE is also plotted.

Table S1. The skin dose, PTD, and AD25 derived from the dose distribution calculated by the SiDE and PHITS.

| Method | Dose index | Value |
| --- | --- | --- |
| PHITS | Skin dose | 10.7 Gy_eq_ |
|  | PTD | 86.9 Gy_eq_ |
|  | AD25 | 72.5 mm |
| SiDE | Skin dose | 9.10 Gy_eq_ |
|  | PTD | 87.7 Gy_eq_ |
|  | AD25 | 70.9 mm |
| PHITS/SiDE | Skin dose | 1.18 |
|  | PTD | 0.991 |
|  | AD25 | 1.02 |
